# The value of speckle tracking echocardiography in early identification of subclinical cardiac involvement in patients with multiple myeloma: a pilot retrospective study

**DOI:** 10.3389/fmed.2026.1848016

**Published:** 2026-06-26

**Authors:** ZhengShuo Jin, Xuehan Mao, Fan Yu, Yanying Wang, Bianhong Wang, Jingxian Li, Jiao Li, Lihong Li, Yuehua Huang

**Affiliations:** Department of Hematology, Beijing Tsinghua Changgung Hospital, School of Clinical Medicine, Tsinghua Medicine, Tsinghua University, Beijing, China

**Keywords:** amyloidosis, echocardiography, global longitudinal strain, multiple myeloma, speckle tracking technology

## Abstract

**Introduction:**

Multiple myeloma (MM) is frequently associated with cardiac involvement, particularly myocardial amyloidosis. Current gold-standard diagnostic modalities include cardiac MRI, and endomyocardial biopsy. Speckle-tracking echocardiography (STE) enables detection of subclinical left ventricular dysfunction prior to overt structural or functional deterioration. This pilot retrospective study explored the association between GLS and clinically defined cardiac involvement in MM patients, in the absence of gold-standard confirmation.

**Methodology:**

We conducted a retrospective analysis of 20 consecutive MM patients, stratified into two groups: cardiac involvement (CI; *n =* 5) and non-cardiac involvement (Non-CI; *n =* 15). CI was defined using surrogate clinical criteria, documented heart failure and elevated NT-proBNP (age-specific) or unexplained interventricular septal thickness >12 mm. GLS was compared between groups, and the exploratory diagnostic performance of GLS was assessed using ROC analysis. A sensitivity analysis excluded patients who did not strictly meet the objective criteria.

**Results:**

Absolute GLS was significantly lower in the cardiac involvement group (median 9.6% vs. 16.9%, *p* = 0.007). GLS showed a promising area under the ROC curve of 0.980 (95% CI: 0.92–1.00) for identifying clinical cardiac involvement, with an optimal cut-off of −16% (sensitivity 100%, specificity 53.3%). All findings are preliminary due to the small sample size and lack of gold-standard confirmation.

**Conclusion:**

In this study, STE-derived GLS demonstrated promising diagnostic performance for identifying cardiac involvement in MM patients. However, given the small sample size, these findings should be considered hypothesis-generating. External validation in large, prospective, multi-center cohorts is required before any clinical implementation.

## Introduction

Multiple Myeloma (MM) is the second most common hematologic malignancy, characterized by the clonal proliferation of abnormal plasma cells and the secretion of monoclonal immunoglobulins [1]. AL amyloidosis is a plasma cell dyscrasia closely associated with MM. Approximately 5–10% of patients with AL amyloidosis exhibit clinical manifestations of MM, while a similar proportion of MM patients develop concurrent AL amyloidosis [1, 2]. Cardiac involvement in MM may manifest as light-chain cardiac amyloidosis, treatment-related cardiotoxicity, or hyperviscosity syndrome. Among these, light-chain amyloidosis is the most typical and severe form. Its pathological hallmark is the deposition of misfolded monoclonal light chains within the myocardial interstitium, leading to cardiac dysfunction and a notably poor prognosis [3].

Currently, the clinical assessment of cardiac involvement in AL amyloidosis or MM relies primarily on symptomatic evaluation and serum biomarkers such as N-terminal pro-brain natriuretic peptide (NT-proBNP) [4], supplemented by cardiac imaging modalities including echocardiography and cardiac magnetic resonance. Findings may include left ventricular (LV) wall thickening on echocardiography [5].

As a widely used tool for evaluating cardiac structure and function, echocardiography often reveals heart failure with preserved ejection fraction (HFpEF) in the early stages of cardiac amyloidosis [23]. This is attributed to myocardial structural alterations caused by amyloid infiltration, which enhance myocardial contractile efficiency at elevated LV end-diastolic pressures, thereby compensating to maintain ventricular function [6]. In advanced disease, a decline in left ventricular ejection fraction (LVEF) may occur, potentially related to the decompensation of myocardial torsion mechanics [6, 7]. Progression to heart failure with reduced ejection fraction (HFrEF) in these patients typically indicates disease advancement and a worsened prognosis. In early cardiac amyloidosis, LVEF often remains preserved until late stages due to concentric hypertrophy and compensatory mechanisms, rendering it insensitive for detecting subclinical myocardial dysfunction [8]. Notably, cardiac amyloidosis can occasionally present with normal wall thickness; such patients usually have mild amyloid infiltration, where LV thickness, though increased from baseline, remains within the conventional echocardiographic normal range [9]. This frequently leads to missed opportunities for early intervention.

In recent years, the development of Speckle-Tracking Echocardiography (STE) has provided a revolutionary non-invasive means of assessing myocardial mechanics. By tracking the motion of acoustic speckles, STE enables the quantitative analysis of myocardial strain in multiple dimensions, including longitudinal and radial directions. Among these parameters, Global Longitudinal Strain (GLS) has proven to detect early myocardial functional impairment [10]. This notion is further supported by a recent state-of-the-art review, which identified cardiac imaging parameters such as GLS as an “emerging approach” with promise for refining prognostic models in the contemporary therapeutic era for AL amyloidosis [11].

In cardiac amyloidosis, studies have demonstrated that GLS impairment precedes the decline in LVEF and exhibits a characteristic pattern of relative apical sparing, which is highly suggestive of the diagnosis [12, 13]. Data indicate that a GLS value ≤16.10% suggests cardiac amyloid involvement with a sensitivity of 93.7% [13]. Traditionally, diagnosis relied on cardiac magnetic resonance with contrast and endomyocardial biopsy, the latter being the gold standard. However, these methods are time-consuming, costly, and impractical for early screening. In contrast, STE, through the assessment of GLS, offers an earlier detection of subtle changes in LV function, providing crucial information for prognostic stratification.

Despite these advances, research systematically applying STE for cardiac assessment in the specific population of MM patients remains limited. There is a paucity of data defining the performance and diagnostic cut-off values of STE parameters for distinguishing MM patients with and without clinical cardiac involvement.

However, to date, no study has systematically evaluated STE-derived GLS in MM patients using only clinically available criteria. This pilot retrospective study therefore aimed to explore the association between GLS and clinically defined cardiac involvement in MM, with explicit acknowledgment of the lack of gold-standard validation and the exploratory nature of the analysis.

## Methods

### Study design and population

This single-center, retrospective, case–control study consecutively enrolled patients with MM who visited the Department of Hematology and completed a standardized cardiac ultrasound assessment between November 2025 and December 2025. Due to the retrospective study design adopted, no patients underwent additional endomyocardial biopsy or cardiac magnetic resonance imaging for this study. Therefore, the cardiac involvement was determined based on the alternative clinical criteria described below.

### Definition of cardiac involvement

**Figure 1:**
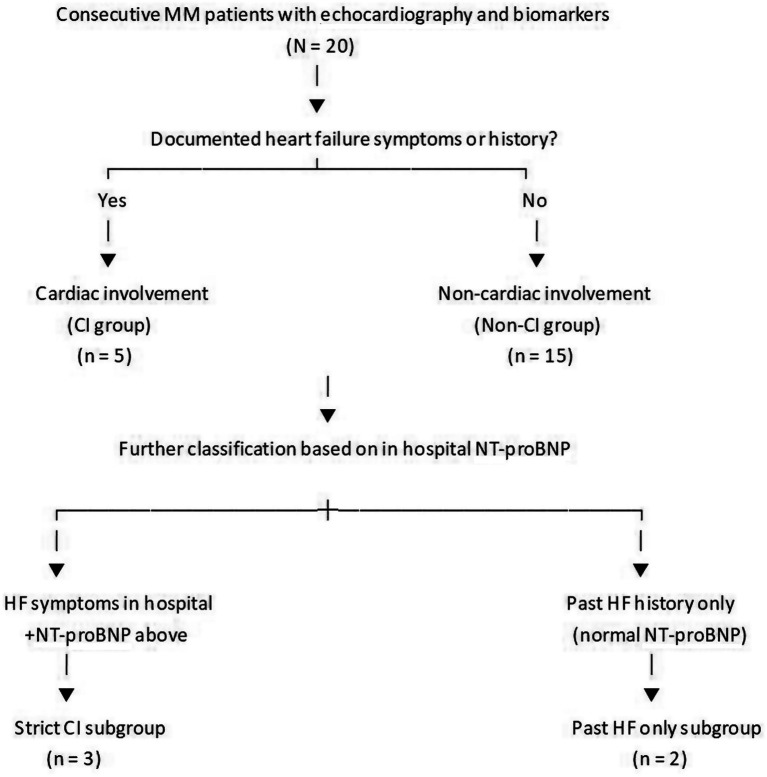
Flowchart of patient selection and group definition. MM, multiple myeloma; CI, cardiac involvement; HF, heart failure; NT-proBNP, N-terminal pro-B-type natriuretic peptide; ULN, upper limit of normal. The CI group was defined by documented heart failure symptoms/history (*n =* 5). Among them, 3 patients also had in-hospital heart failure with NT-proBNP above the age-specific upper limit (strict definition), while 2 patients had only a past history of heart failure with normal in-hospital NT-proBNP. No patient underwent biopsy or cardiac MRI.

According to established clinical and diagnostic criteria, patients were divided into two groups: (1) The cardiac involvement group, defined by meeting at least one of the following criteria: Patients with a clinical diagnosis of heart failure but neither elevated NT-proBNP nor increased IVST were not excluded from the primary analysis; (2) the non-cardiac involvement group, consisted of patients without any clinical diagnosis of heart failure and with normal NT-proBNP and normal wall thickness. Baseline patient data were retrospectively collected through the electronic medical record system, encompassing demographic information, MM-related staging and classification, past medical history, and examination data relevant to this study (Figure 1). Echocardiographic parameter measurements for all patients followed the guidelines of the American Society of Echocardiography, including measurement of LVEF and interventricular septal thickness (IVST) at end-diastole. For speckle-tracking analysis, high-frame-rate two-dimensional dynamic images of at least three consecutive cardiac cycles were acquired and stored from the apical four-chamber, two-chamber, and three-chamber views. The software automatically tracked myocardial acoustic speckles to generate left ventricular GLS, with the GLS value being the average strain of all segments across the three views. All analyses were performed by a senior cardiovascular ultrasound physician. Fasting venous blood samples were collected from patients concurrently and test the indicators related to cardiac injury, which included Myoglobin (MYO), High-sensitivity cardiac Troponin I (hs cTnI), Creatine Kinase-Myocardial Band (CK-MB), N-terminal pro-B-type Natriuretic Peptide (NT-proBNP). All testing was completed in the central laboratory of our hospital following the manufacturer’s instructions.

### Statistical analysis

Statistical analysis was performed using IBM SPSS Statistics31.0. Continuous variables conforming to a normal distribution are expressed as mean ± standard deviation and compared between groups using the independent samples *t*-test; variables not conforming to a normal distribution are expressed as median and compared using the Mann–Whitney U test. Categorical variables are presented as frequencies and compared between groups using the chi-square test or Fisher’s exact test. The Shapiro–Wilk test was used to assess the normality of variables. Spearman’s rank correlation analysis was employed to explore the association between cardiac imaging parameters and serological biomarkers. The diagnostic efficacy of each indicator for cardiac involvement was evaluated by plotting receiver operating characteristic (ROC) curves, calculating the area under the curve (AUC), optimal cutoff value, sensitivity, and specificity. Given the small sample size, the reported AUC and cutoff values should be interpreted as preliminary. All statistical analyses were two-sided, with a *p* < 0.05 considered statistically significant.

## Results

### Baseline characteristics

A total of 20 patients with multiple myeloma were enrolled in this study, comprising 5 patients in the cardiac involvement group and 15 patients in the non-cardiac involvement group. Baseline demographics, MM subtype, ISS stage, and cardiovascular risk factors (hypertension, diabetes, hyperlipidemia) did not differ significantly between groups (all *p* > 0.05). Retrospective chart review of the 5 CI patients showed that all had a documented history of heart failure symptoms (Table 1). Among them, 3 patients developed heart failure symptoms during hospitalization with NT-proBNP above the age-specific upper limit. The other 2 patients had only a past history of heart failure but normal or borderline NT-proBNP during hospitalization (Supplementary Table S1).

**Table 1:** Baseline characteristics of patients with multiple myeloma.

Variable	Non-cardiac involvement (non-CI; *n =* 15)	Cardiac involvement (CI; *n =* 5)	*p*-value
Age	66.73 ± 2.56	75 ± 3.85	0.114
Sex			0.2
Male	6	5	
Female	9	0	
Total	15	5	
MM type			0.18
IgG	7	0	
IgA	4	2	
IgE	0	0	
IgD	1	0	
Light chain	3	3	
Total	15	5	
ISS stage			0.047
Stage I	4	0	
Stage II	3	4	
Stage III	8	1	
Total	15	5	
Past medical history			
High blood pressure	3	8	0.795
Diabetes	2	7	0.371
Coronary Artery Disease	3	3	0.371

### Comparison between groups

We performed statistical analyses on collected markers of cardiac structure and function to compare the differences between speckle-tracking echocardiography, conventional echocardiography, and serological cardiac biomarkers in identifying cardiac involvement in patients with multiple myeloma. The parameters collected included IVST, LVEF, GLS, NT-proBNP, and hs cTnI. The results showed no significant difference in IVST between the non-cardiac involvement group and the cardiac involvement group (median 10 mm vs. median 11 mm, *p* > 0.05). Similarly, there was no significant difference in LVEF between the two groups (59.37 ± 16.37% vs. 57.20 ± 8.78%, p > 0.05). Regarding cardiac function, although no significant differences were found in LVEF or IVST, the absolute value of GLS was significantly lower in the cardiac involvement group (9.6% vs. 15.9%, *p* < 0.05). In terms of serum biomarkers, the median levels of both NT-proBNP and hs-cTn were significantly higher in the cardiac involvement group compared to the non-cardiac involvement group (Table 2).

**Table 2:** Comparison of echocardiographic parameters and serum biomarkers.

Variable	Non-cardiac involvement (Non-CI; *n =* 15)	Cardiac involvement (CI; *n =* 5)	*P*-value
Echocardiographic parameters			
LVEF%	59.37 ± 16.37	57.2 ± 8.78	0.7
IVST, mm	10 (1)	11 (5)	0.63
Speckle-tracking parameters			
GLS,%	15.9 (5.6)	9.6 (7.1)	0.007
LVEF/GLS	3.646 (0.736)	5.43 (8.29)	0.002
Myocardial injury biomarkers			
hs cTnI (ng/L)	2.4 (3.1)	13.6 (80.4)	0.3
NT-proBNP (pg/mL)	115.8 (403.38)	1139.88 (7844)	0.4
MYO	27.29 ± 13.75	72.27 ± 50.6	0.118
CKMB	1.46 ± 0.843	1.67 ± 0.814	0.63

### Correlation analysis

**Figure 2:**
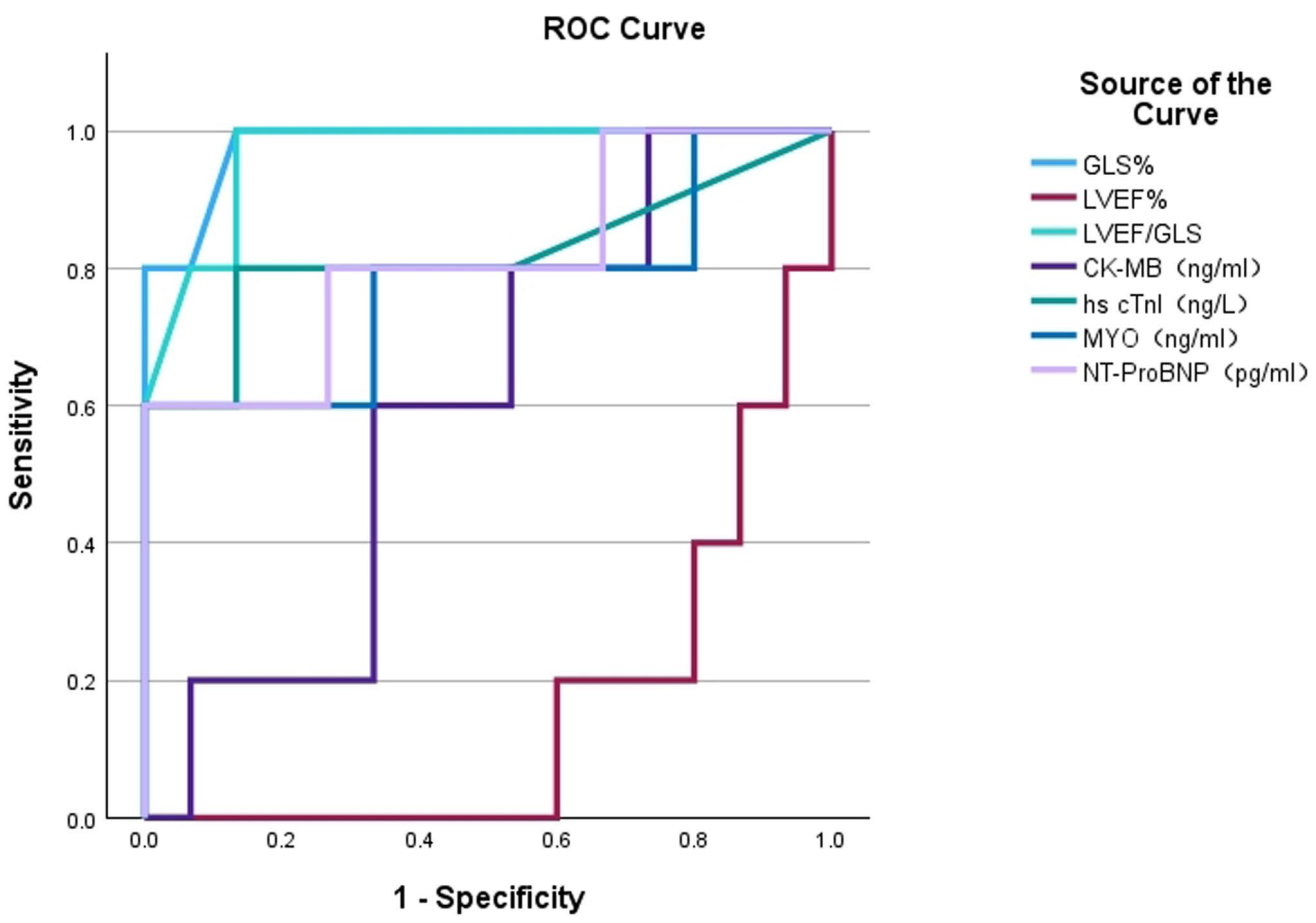
Correlation analysis of various cardiac indicators. To evaluate the diagnostic value of different indicators for identifying cardiac involvement in patients with multiple myeloma, we constructed ROC curves for each parameter. The analysis of the AUC revealed that GLS demonstrated the highest diagnostic performance, with an AUC of 0.980 (95% CI: 0.934–1.000). At a cut-off value of −16%, GLS achieved a sensitivity of 100% and a specificity of 53.3% for diagnosis. In contrast, the AUC for Left Ventricular Ejection Fraction (LVEF) was only 0.227 (95% CI: 0.043–0.411), and the AUC for serum NT-proBNP was 0.813 (95% CI: 0.617–1.000).

Spearman correlation analysis performed on the entire cohort (*n =* 20) did not reveal a statistically significant linear correlation between absolute GLS and key myocardial injury biomarkers, including hs-cTnI, NT-proBNP, and MYO (all *p* > 0.05). To investigate further, we conducted a subgroup analysis specifically within the cardiac involvement group (*n =* 5). Within this subgroup, GLS demonstrated a strong trend of negative correlation with several myocardial injury markers: a perfect negative correlation with CK-MB (rs = −1.000, *p* = 0.025), and negative correlations with hs-cTnI (rs = −0.700) and NT-proBNP (rs = −0.500). These correlation coefficients, especially the perfect correlation with CK-MB, should be interpreted with great caution given the extremely small sample size (*n =* 5) and the presence of potential outliers. Nevertheless, these preliminary observations suggest that in patients with clinically defined cardiac involvement, deterioration of myocardial function may be accompanied by concomitant changes in serological markers of myocardial injury. However, this hypothesis requires validation in larger, independent cohorts (Figure 2).

### Diagnostic performance of GLS and other parameters

**Figure 3:**
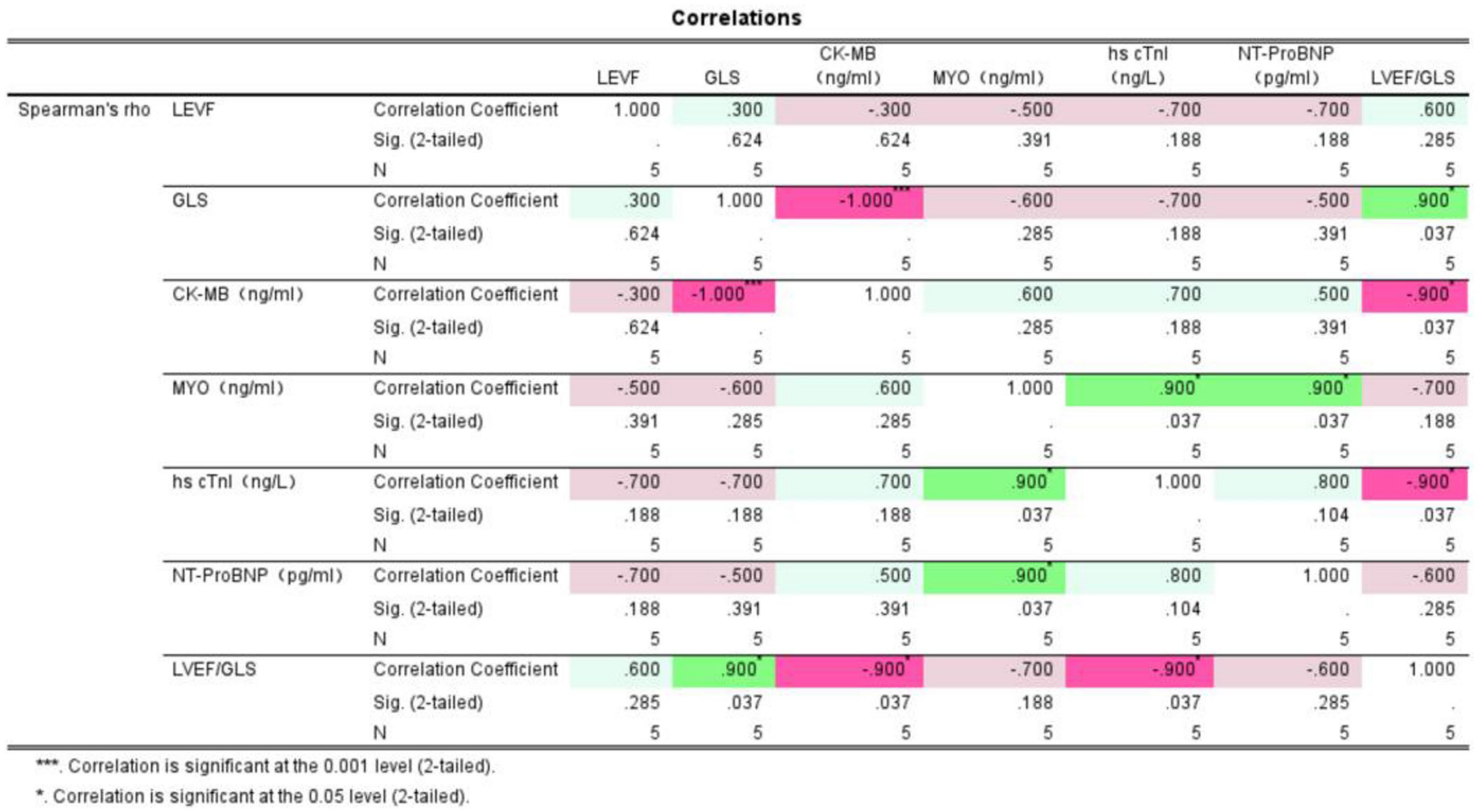
Result of ROC curve. Receiver operating characteristic (ROC) curves of GLS, conventional echocardiographic parameters, and serum biomarkers for identifying clinically defined cardiac involvement in MM patients (CI *n =* 5, Non-CI *n =* 15).

To evaluate the exploratory diagnostic value of different indicators for identifying clinically defined cardiac involvement in patients with multiple myeloma, we constructed receiver operating characteristic (ROC) curves for each parameter (Figure 3). The analysis of the area under the curve (AUC) revealed that GLS demonstrated the diagnostic performance among the tested parameters, with an AUC of 0.980 (95% confidence interval [CI]: 0.934–1.000) with an optimal cut-off of −16% (sensitivity 100%, specificity 53.3%). In contrast, the AUC for left ventricular ejection fraction (LVEF) was only 0.227 (95% CI: 0.043–0.411), indicating no discriminative ability, and the AUC for serum NT-proBNP was 0.813 (95% CI: 0.617–1.000).

## Discussion

### Clinical background and rationale

Multiple Myeloma can be complicated by amyloidosis affecting various systems and organs. Approximately 20% of patients with MM progress to AL amyloidosis due to the pathophysiological nature of their disease; conversely, only about 10% of patients with systemic amyloidosis meet the diagnostic criteria for MM [6, 26]. MM often leads to multi-system involvement, yet the clinical manifestations of cardiac amyloidosis are non-specific. Consequently, rates of missed diagnosis, misdiagnosis, and delayed diagnosis are high [24, 25]. This results in many patients presenting with symptoms of heart failure at the time of diagnosis, associated with severe illness and a poor prognosis. The median survival of patients with systemic amyloidosis and cardiac involvement is merely 6 months, with the majority of deaths being cardiac in origin, including sudden death and heart failure [2, 14]. Therefore, enhancing early clinical recognition is paramount. Left ventricular global longitudinal strain (GLS) by speckle-tracking echocardiography (STE) can serve as an early indicator.

### Main findings and comparison with literature

In this study, GLS was significantly reduced in MM patients with cardiac involvement despite preserved LVEF and normal septal thickness, consistent with the known pathophysiology of cardiac amyloidosis. Previous research suggests that a GLS ≤ −16.10% indicates cardiac amyloid involvement with a sensitivity of 93.7%, and an LVEF/GLS ratio >4.95 has a sensitivity of 75% and specificity of 66% [15, 16]. This may reflect the decoupling of cardiac ejection function from myocardial contractile function during disease progression. In early amyloidosis, heart failure with preserved ejection fraction (HFpEF) can occur, where GLS is significantly impaired while LVEF may be compensatorily preserved, leading to an increase in this ratio [13].

### Comparison with conventional echocardiography and biomarkers

Echocardiography, being the most widely used technique in clinical practice for cardiac function assessment, was included in the analysis as a reference benchmark. This allows for a quantitative comparison of the diagnostic advantage of the novel STE technique (via GLS) over traditional standard methods. Similarly, NT-proBNP is a classic serological biomarker reflecting ventricular wall stress and cardiac load, and is a key tool for the diagnosis, staging, and prognostic evaluation of heart failure [17, 18]. Its inclusion in the comparison aimed to explore whether non-invasive imaging indicators might provide diagnostic information superior or equivalent to serological markers. The LVEF/GLS ratio used in this study is a composite parameter. Previous research suggests that a GLS ≤ 16.10% indicates cardiac amyloid involvement with a sensitivity of 93.7%; while an LVEF/GLS ratio >4.95 has a sensitivity of 75% and a specificity of 66% for diagnosing cardiac amyloidosis [15, 16]. This may reflect the decoupling of cardiac ejection function from myocardial contractile function during disease progression. In the early stages of amyloidosis, heart failure with preserved ejection fraction (HFpEF) can occur, where GLS is significantly impaired while LVEF may be compensatorily preserved, leading to an increase in this ratio [13]. Therefore, this ratio might amplify the signal of this “functional mismatch.” We found that GLS abnormalities occurred independently of LVEF, which aligns with the early clinical-pathological features of cardiac amyloidosis: amyloid deposition in the myocardial extracellular matrix primarily impairs diastolic function and longitudinal contractility, subtle changes that GLS can sensitively capture [19, 27]. In contrast, LVEF, as an indicator measuring global ventricular volumetric change, remains preserved in early disease due to compensatory mechanisms, declining only in the terminal stage when the myocardium is extensively involved [20]. These results resonate with previous studies in AL amyloidosis, which also confirmed GLS as an earlier and more sensitive marker of myocardial dysfunction than LVEF [21].

### Implications for prognostic models

Our findings are consistent with the current literature for refining prognostic models in AL amyloidosis. It emphasized that older risk stratification models, developed in eras with less effective therapies, have become disconnected from actual outcomes, and that there is an urgent need for validation studies incorporating novel markers [11]. Our pilot data, despite their preliminary nature, directly respond to this call by providing early evidence supporting the potential utility of GLS in MM patients with suspected cardiac involvement, GLS showed a promising exploratory signal.

### Correlations with serum biomarkers

The observed correlation pattern between GLS, serum hs-cTnI and NT-proBNP in this study was “overall no significant difference, but strong correlation in different subgroups”, it’s provides profound insights into the disease progression of cardiac involvement in multiple myeloma. hs-cTnI and NT-proBNP, respectively, reflect myocardial cell damage and myocardial wall pressure, and are reliable and effective biomarkers for assessing the stage and prognosis of amyloidosis [22]. The absence of significant correlations across the entire cohort may indicate an early subclinical phase, during which microscopic alterations in myocardial structure and mechanics have already occurred, insufficient myocardial necrosis or substantial elevation in pressure overload has taken place to elicit marked increases in serum biomarker levels. Our subgroup analysis suggests a hypothesis that in patients with confirmed cardiac involvement, myocardial strain rates may correlate with biomarkers of injury. However, we reiterate that these subgroup correlations are derived from small sample, they should be considered hypothesis-generating. Thus, myocardial strain rate may lie further upstream in this continuum, acting as a marker of “functional impairment,” whereas hs-cTn/NT-proBNP more strongly indicate the exacerbation of “injury and stress.”

Furthermore, during our research, we found that more than two patients did not exhibit any clinical symptoms of heart failure, such as chest tightness or breathing difficulties, during the diagnosis and treatment of multiple myeloma. The markers of myocardial injury and NT-proBNP levels did not show significant increases. Routine echocardiography did not reveal signs of ventricular septal thickening or reduced left ventricular ejection fraction. However, the ST examination revealed characteristic patterns of protection at the apex and reduced myocardial diastolic function, which alerted us to the possibility of myocardial involvement. Subsequently, these patients underwent cardiac MRI, but no typical late gadolinium enhancement pattern was observed. Nevertheless, based on the ST examination results, we adjusted the treatment plan for these patients, including reducing drugs toxic to the heart, strengthening fluid balance management, closely monitoring parameters related to cardiac function, and taking cardiac protection meas. Based on the preliminary results of this study, we suggest that STE should be considered for baseline and follow-up assessments in patients with multiple myeloma, but this recommendation requires validation in larger studies before routine clinical adoption.

## Limitations

There are also several limitations in this pilot study. The sample size is small (*N =* 20, only 5 in the CI group), which is prone to optimization bias. A single variable like GLS might achieve near perfect separation by chance. Consequently, the reported AUC of 0.980 and the cutoff of −16% are likely overestimates. The low specificity (53.3%) at this cutoff is likely related to the small sample size; therefore, this result should be viewed as a preliminary hypothesis to guide future in-depth research. The definition of cardiac involvement was heterogeneous and incorporated NT proBNP and echocardiographic wall thickness, which were also examined as diagnostic markers, introducing a risk of circular bias. Although a sensitivity analysis excluding patients diagnosed solely by noninvasive criteria yielded similar results, this does not fully eliminate the concern. Moreover, no patient underwent endomyocardial biopsy or cardiac MRI as a gold standard.

## Conclusions and future directions

Based on these preliminary results, we suggest that STE should be considered for baseline and follow-up assessments in MM patients, but this recommendation requires validation in larger studies before routine clinical adoption. Our results are derived from a single center and lack external validation, its primary value is to generate hypotheses for future research, not sufficient to become definitive diagnostic criteria at this present stage. Future work should include prospective studies with gold-standard definitions and longitudinal designs to assess whether dynamic GLS changes can predict clinical heart failure events.

## Data Availability

The original contributions presented in the study are included in the article/Supplementary material, further inquiries can be directed to the corresponding author.
