# Supplementary material for: The value of speckle tracking echocardiography in early identification of subclinical cardiac involvement in patients with multiple myeloma: a pilot retrospective study
Source: Front Med (Lausanne). 2026 Jun 26;13:1848016. doi: 10.3389/fmed.2026.1848016 (PMC13367762; doi:10.3389/fmed.2026.1848016)
Supplement: Supplementary file 1 [file Supplementary_File_1.DOCX]

**S.Table 1. Individual characteristics of the 5 patients in the cardiac involvement group (CI)**

| Patient | Sex | age | HF symptoms | MM Classification | ISS Classification | IVST(mm) | LEVF | GLS% | CK-MB（ng/ml） | MYO（ng/ml） | hs cTnI（ng/L） | NT-ProBNP（pg/ml） | LVEF/GLS |
| --- | --- | --- | --- | --- | --- | --- | --- | --- | --- | --- | --- | --- | --- |
| 1 | M | 78 | yes | Light chain | II | 10 | 42 | 9.6 | 1.6 | 136.47 | 145.7 | 14204 | 4.375 |
| 5 | M | 69 | yes | Light chain | II | 13 | 58 | 14 | 3.02 | 76.77 | 23.3 | 1140 | 4.055 |
| 7 | M | 78 | yes | Light chain | II | 11 | 64 | 9.1 | 1.22 | 27.25 | 6.2 | 75.29 | 5.66 |
| 13 | M | 86 | yes | IgA | II | 8 | 60 | 1.5 | 0.88 | 16.89 | 2 | 419.6 | 19.3 |
| 15 | M | 64 | yes | IgA | III | 14 | 62 | 10.9 | 1.64 | 104 | 13.6 | 1980 | 5.43 |
|  |  |  |  |  |  |  |  |  |  |  |  |  |  |

Abbreviations: HF, heart failure; NT, NT‑proBNP; ULN, upper limit of normal.
